# Does age acquired immunity confer selective protection to common serotypes of *Campylobacter jejuni*?

**DOI:** 10.1186/1471-2334-5-66

**Published:** 2005-08-23

**Authors:** Gordon Miller, Geoff M Dunn, Thomas MS Reid, Iain D Ogden, Norval JC Strachan

**Affiliations:** 1 School of Biological Sciences, University of Aberdeen, Cruickshank Building, Aberdeen, UK; 2 School of Engineering and Physical Sciences, Department of Engineering, University of Aberdeen, Fraser Nobel Building, Aberdeen, UK; 3 Microbiology Department, Aberdeen Royal Infirmary, Aberdeen, UK; 4 Department of Medical Microbiology, University of Aberdeen, Foresterhill, Aberdeen, UK

## Abstract

**Background:**

*Campylobacter *infection is a major cause of bacterial gastrointestinal disease. Exposure to *Campylobacter *is known to produce an immune response in humans that can prevent future symptomatic infections. Further, studies of the general population have shown that seroprevalence to *Campylobacter *increases with age.

**Methods:**

A large collection of serotyped *Campylobacter *isolates, obtained from human clinical faecal samples, were analysed by comparing the ratio of uncommon to common serotypes by different age groups, using χ^2 ^tests.

**Results:**

We have identified that older age groups, as well as having generally lower incidence, are significantly less likely to be infected by the more common serotypes.

**Conclusion:**

These results are indicative of acquired immunity, however, further studies are needed to rule out the confounding effects of the variations in exposure pathways experienced by different age groups.

## Background

*Campylobacter *is the greatest cause of human bacterial gastroenteritis in the developed world [1], with more than 50,000 cases reported in the UK alone each year [2]. However, due to under-reporting, it is believed that the true number of cases is around eight times this [2], suggesting humans are infected on average at least once during their lifetime. Symptoms of campylobacteriosis include fever, abdominal cramp, and bloody diarrhoea, which can last for approximately seven days after infection, with one in a 1000 cases leading to the more serious neurological disease, Guillain-Barré syndrome [3].

Approximately 90% of *Campylobacter *infections are *C. jejuni*, while the remainder are predominantly *C. coli *[4]. A variety of typing techniques have been used to subtype these species, including phenotypic Penner heat stable antigen serotyping, genotypic PFGE, AFLP and MLST, which have demonstrated high variability in the genome.

Human seroprevalence studies of *C. jejuni *specific antigens have shown that IgG, IgM, and IgA antibodies are produced during infection [5,6]. The IgM and IgA antibody levels quickly decrease after infection, however, the IgG antibodies can remain elevated for months or years afterwards [5]. A recent study of seroprevalence in Danish adults looked at IgG antibodies in a sample of 1112 people from Copenhagen [7]. It was found that the percentage of people with *C.jejuni*-specific IgG antibodies increased with age, from 20.6% in the 15–34 years age group to 32.4% in the 50 – 69 years age group.

Humans are likely to be exposed to *Campylobacter *a number of times during their lifetime, resulting in immune responses that can last several years [8]. Further, as the types/strains of *Campylobacter *are diverse, it is probable that people are more likely to develop immune protection to the more common types to which they are exposed. Hence we hypothesise that the general population develops increased immunity to the more common types of *Campylobacter *as they grow older. Here we test this hypothesis by analysing a large dataset of Penner serotyped cases of human campylobacteriosis, stratified by age, to determine if the prevalence of common serotypes decreases with the age of the population.

## Methods

Data were collated from the Grampian NHS trust reporting human infections of *C. jejuni *in Grampian, Scotland (approximately 2300 cases during 1997 – 1999). These data included each patient's age, gender and date of infection. Isolate serotyping had been performed on these cases, using the Penner method [9]. Cases with no recorded age (105), as well as untyped and *C. coli *cases (168), were omitted from the data set, leaving 1955 typed cases of *C. jejuni*.

The *C. jejuni *cases were separated into 48 Penner serotypes, which were then collected together into recognised groupings, such as HS:1,44, HS:6,7, and HS:4 complex (comprising serotypes HS:4, 13, 16, 43, 50) [10]. The prevalence of each serotype was determined, with serotypes accounting for more than 10% of the cases being defined as common, and those accounting for less than 10% being classed as uncommon. These data were stratified by age (five year age groups), then ratios of uncommon to common types were determined. The incidence (cases per 100,000) of each age group was calculated, using the population data from the 2001 Scottish Census [11]. Finally χ^2 ^tests were used to test the statistical significance of differences between age group ratios.

## Results

**Figure 1:**
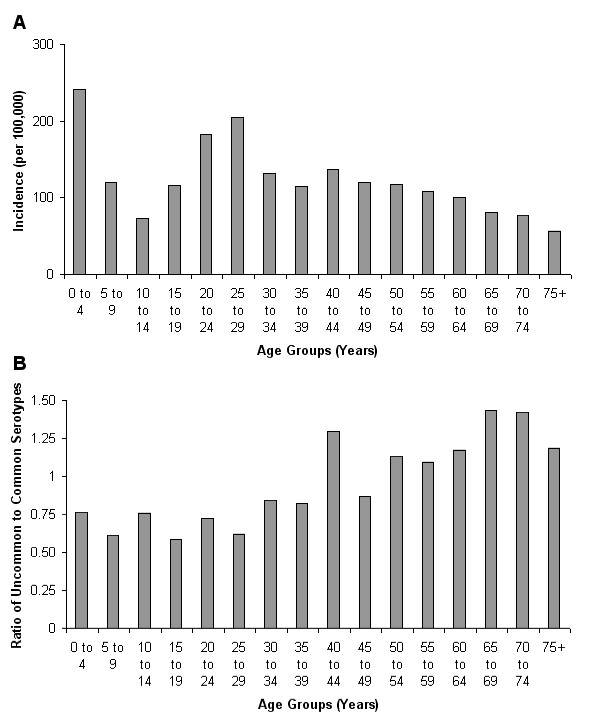
***C. jejuni *incidence and ratio of uncommon to common serotypes**. Human campylobacteriosis in Grampian Scotland (1997–1999). a) Incidence of reported cases and b) Ratio of uncommon to common serotypes.

The overall incidence of *Campylobacter *infection by five-year age groups (Figure 1A) shows a large number of cases in the 0–4 years age group (241 cases per 100,000). There is a drop off, before a second peak found in the 25–29 years age group (205 cases per 100,000). From 30 years of age onwards there is a gradual reduction in cases down to the minimum of less than 60 cases per 100,000 among the patients 75 years and older.

The most prevalent Penner serotypes found in these data were HS:4 complex, HS:2, and HS:1,44 (427, 420, and 205 cases respectively) which account for a total of 53.8% of all tested cases (Table 1). These three type groups were classified as common, for comparison against the 40 other uncommon serotypes.

**Table 1:** *C. jejuni *Serotypes Number of reported cases of *C. jejuni *for Grampian, Scotland (1997–1999) by Penner serotypes, including separation into groups less than and greater than 40 years of age.

Penner Serotype	Total (%)	Age	
		<40 (%)	>40 (%)
4 complex^a^	427 (21.8)	286 (23.8)	141 (18.7)
2	420 (21.5)	285 (23.8)	135 (17.9)
1,44	205 (10.5)	130 (10.8)	75 (9.9)
11	128 (6.5)	55 (4.6)	73 (9.7)
6,7	113 (5.8)	67 (5.6)	46 (6.1)
8	45 (2.3)	18 (1.5)	27 (3.6)
23	44 (2.3)	25 (2.1)	19 (2.5)
21	27 (1.4)	16 (1.3)	11 (4.5)
5	26 (1.3)	17 (1.4)	9 (1.2)
9	23 (1.2)	14 (1.2)	9 (1.2)
37	23 (1.2)	10 (0.8)	13 (1.7)
Other^b^	474 (24.2)	277 (23.1)	197 (26.1)
Total	1955	1200	755

^a^Serotypes 4, 13, 16, 43, 50

^b ^Types accounting for less than 1% of total.

The ratios of uncommon to common types by five-year age groups (Figure 1B) show that for ages 0 – 39 there is a consistently greater number of infections from the common types (HS:4 complex, HS:2, HS:1,44), with an uncommon/common mean ratio of 0.71 (± 0.10). For patients aged 40 years and older, all age groups but one (45–49 years) have a higher incidence of uncommon types (mean: 1.20 ± 0.18). A χ^2 ^test confirms that the difference is highly statistically significant (p < 0.001).

## Discussion

In this study we compiled the Penner serotype data for nearly 2000 cases of human *Campylobacter jejuni *infections in Grampian, Scotland, determined which were the common types, and identified that these types are significantly more prevalent in younger age groups (less than approximately 40 years). The reduced frequency of common types in older age groups, along with a general reduction in incidence of infection, provides evidence for the hypothesis that people develop immunity to the most common types of *C. jejuni *during their lifetimes.

From the age-specific incidence data (Figure 1A) it is clear that the highest incidence occurs in the 0–4 years age group, which agrees with previous studies [12]. This could be due to many factors including lack of immunity, higher rates of reporting in the age group, or a higher risk of exposure to a particular infection pathway. For example, in rural regions such as Grampian, Scotland, where farm animals shed high loads into the environment, small children may have a high likelihood of coming into contact with, and ingesting, the bacteria. The reduction of incidence in the 5–9 and again in the 10–14 age groups is similar to previous studies [13], which may be due to improved hygiene reducing the risk of environmental exposure. This trend then reverses with a sharp increase in incidence in the older teens/early twenties. This occurs around the age when people are more likely to be travelling and hence coming into contact with new strains. Case-control studies have found that international and national travel is an important risk factor for infection [14]. In humans >30 years, a gradual reduction in incidence is observed. This, taken together with the results of the Danish study [7] showing that seroprevalence increases with age, and the fact that it has been demonstrated that elevated levels of *Campylobacter jejuni *immunoglobulin A reduces the risk of *Campylobacter *diarrhoea in adults traveling to Thailand [15], suggests that immunity may be causing protection from *Campylobacter *infection, or at least symptomatic infection. However, the lower incidence could also be caused by reduced exposure to the infection pathways more common in younger age groups.

The common serotypes found in these data (Table 1) are consistent with those found in previous studies [16,17], which are also the same types found in poultry and farm animals [17]. Although we do not have the epidemiological data to ascertain whether the human cases can be attributed to animals/poultry it is clear that the majority of cases in all age groups below 40 are from these common serotypes (Figure 1B).

## Conclusion

These data presented here demonstrate lower incidence along with the reduced occurrence of infection from the common serotypes with age, supporting the hypothesis of increased immunoprotection in the population with age. However, it is possible that changing exposure to *Campyolobacter *with age would confound our results, which can only be clarified by performing a comprehensive epidemiological age-stratified exposure assessment.

## Competing interests

The author(s) declare that they have no competing interests.

## Authors' contributions

GM collected the data, performed the analysis, and drafted the manuscript. GMD participated in the design of the study and helped draft the manuscript. TMSR carried out the serotyping and helped collect the data. IDO participated in the design of the study and helped in the interpretation of the data. NJCS conceived of the study, coordinated it, and assisted with the statistical analysis.

## Pre-publication history

The pre-publication history for this paper can be accessed here:


